# Family-based GWAS for dental class I malocclusion and clefts

**DOI:** 10.1186/s12903-024-04444-x

**Published:** 2024-06-07

**Authors:** Mariana Bezamat, Chelsea E. Carver, Alexandre R. Vieira

**Affiliations:** 1 Department of Oral and Craniofacial Sciences, University of Pittsburgh School of Dental Medicine, Pittsburgh, PA USA; 2 School of Dental Medicine, East Carolina University, Greenville, NC 27834-4354 USA

**Keywords:** Class I malocclusion, Cleft lip, Cleft palate, Genetic variation, GWAS

## Abstract

**Background:**

Individuals born with cleft lip and/or palate who receive corrective surgery regularly have abnormal growth in the midface region such that they exhibit premaxillary hypoplasia. However, there are also genetic contributions to craniofacial morphology in the midface region, so although these individuals appear to have Class III skeletal discrepancy, their molar relationship may be Class I. Past genome-wide association studies (GWASs) on skeletal Class II and III malocclusion suggested that multiple genetic markers contribute to these phenotypes via a multifactorial inheritance model, but research has yet to examine the genetic markers associated with dental Class I malocclusion. Thus, our goal was to conduct a family based GWAS to identify genes across the genome that are associated with Class I malocclusion, as defined by molar relations, in humans with and without clefts.

**Methods:**

Our cohort consisted of 739 individuals from 47 Filipino families originally recruited in 2006 to investigate the genetic basis of orofacial clefts. All individuals supplied blood samples for DNA extraction and genotyping, and a 5,766 single nucleotide polymorphism (SNP) custom panel was used for the analyses. We performed a transmission disequilibrium test for participants with and without clefts to identify genetic contributors potentially involved with Class I malocclusion.

**Results:**

In the total cohort, 13 SNPs had associations that reached the genomic control threshold (*p* < 0.005), while five SNPs were associated with Class I in the cohort of participants without clefts, including four associations that were identified in the total cohort. The associations for the SNPs *ABCA4* rs952499, *SOX1-OT* rs726455, and *RORA* rs877228 are of particular interest, as past research found associations between these genes and various craniofacial phenotypes, including cleft lip and/or palate.

**Conclusions:**

These findings support the multifactorial inheritance model for dental Class I malocclusion and suggest a common genetic basis for different aspects of craniofacial development.

## Introduction

The “father of modern orthodontics”, Dr. Edward Hartley Angle determined three types of malocclusion: Class I, II, and III [1]. In the present study, we are interested in the Class I molar classification or “neutroclusion”, which is established as the mesiobuccal cusp of the maxillary first molar occluding with the buccal grove of the mandibular first molar [1, 2]. It has been suggested that 92% of malocclusion cases have unknown etiology and genetics may play a significant role in craniofacial morphology of the midface region [2–4].

Cleft lip and/or palate is a common condition that affects about 1 in every 600 newborn babies. It consists of a failure in the closure of fetal facial tissues, including lip and/or palate tissues, prenatally [5]. Individuals born with cleft lip and/or palate who receive corrective surgery regularly have disrupted growth in the midface region such that they may exhibit premaxillary hypoplasia, crossbites, and/or malocclusion [6, 7].

Past genome-wide association studies (GWASs) on skeletal Class II and III malocclusion suggested that multiple genetic markers contribute to these phenotypes via a multifactorial inheritance model [4], but research has yet to examine the genetic markers associated with dental Class I malocclusion in individuals with or without clefts.

In the present study, we conducted a family-based GWAS in individuals affected by dental Class I malocclusion, as defined by molar relations, and clefts. We aimed to identify genes across the genome that are associated with Class I malocclusion in 47 Filipino families in order to gain insight into the genetic basis of this favorable craniofacial phenotype in humans with and without clefts.

## Methods

### Participants

Our study cohort consisted of 739 individuals (377 males and 362 females) from 47 families who were small-scale fishermen or landless rural dwellers residing in the central region of the Philippines, mainly Cebu Island, and the surrounding islands. This cohort was previously obtained for the purpose of investigating orofacial characteristics and genetic profiles of individuals who had similar cultural backgrounds and who were exposed to similar environmental factors [8]. Since the individuals in the cohort had shared variables of similar cultural backgrounds and the same area of residence, their access to care and their environmental influences were also similar, reducing possible confounders.

The original phenotype examined in the cohort was orofacial clefts. Informed consent was obtained from all participants and the project had appropriate approvals from Institutional Review Boards. All participants supplied blood samples for DNA extraction and genotyping and underwent a clinical evaluation by an experienced dentist to assess whether they had Class I malocclusion. The clinical criterion for making the diagnosis was a Class I molar relationship based on a visual clinical examination of the individual’s molar-canine relationships where the mesiobuccal cusp of the maxillary first molar should be occluding with the buccal grove of the mandibular first molar [2].

One hundred and nine individuals in the cohort were diagnosed with Class I malocclusion (55 males and 54 females). In the present study, 90 of the 739 individuals included in the analysis had clefts and only six individuals were diagnosed with Class I malocclusion and clefts concurrently. There was not a large diversity of ages within the sample population. Most of the participants were young, in their twenties, after facial growth is complete. Further oral conditions that were identified in this population and are common in the general population include gingivitis, plaque, dental caries, and torus palatinus.

### Genomic analyses

A custom panel including 5,766 single nucleotide polymorphisms (SNPs) was previously used to generate genotyping data at the Center for Inherited Disease Research (CIDR). The criteria for selecting the custom panel included the minor allele frequency of the SNP, the location of the SNP in the gene, the postulated function of the gene, and the linkage disequilibrium structure of a genomic region that accounts for proximate genes. The PedCheck program was utilized to test for inconsistencies in the data due to non-paternity or other errors [9].

In the present study, we performed a family-based transmission disequilibrium test (TDT) using PLINK software [10], which tested for the over-transmission of alleles of the 5,766 markers and Class I malocclusion in the cohort. Since the TDT measures the transmission of marker alleles of heterozygous parents to the affected offspring and the non-transmitted alleles act as controls to the transmitted ones [11], the test is suitable and can avoid the issue of potential deceptive associations that can be present in case-control studies. We conducted data analysis in the total sample and in a subset of the sample that excluded individuals with clefts, as both Class I malocclusion and clefts affect the same or adjacent craniofacial structures, and the additional analysis could distinguish genetic contributors that also contribute to clefts. In addition, we generated adjusted significance values to account for multiple testing. Since the Bonferroni correction and the false discovery rate (FDR) correction were too stringent, we also report the genomic control (GC) correction to set our significance threshold of *p* < 0.005. Given our limited sample of individuals with Class I (109 people) and because we did not want to risk not identifying relevant biological associations for this understudied phenotype, we report GC (a more relaxed correction for multiple testing, *p* < 0.005). In the present study, the Bonferroni correction sets the significance threshold to *p*-value < 8.7 × 10^− 6^.

Additional analyses for sex differences and Class I phenotype presence (affected and unaffected) according to each genotype were performed for the most relevant results found in the aforementioned TDT analysis (GC *p* < 0.005).

## Results

Following the completion of the TDT for the 47 families in the total cohort, we discovered that there were relevant SNPs (GC corrected *p* value < 0.005) present in chromosomes 1, 3, 4, 5, 10, 11, 12, 13, 14, 15, 20, and 23. The SNPs with the lowest GC corrected *p* values associated with increased likelihood of Class I malocclusion development were as follows: rs952499 (*ABCA4*), rs3847993 (*IL17D*), and rs726455 (*SOX1-OT*) (Table 1). When we completed the TDT tests in the subset of the sample excluding individuals with clefts, only four of the associations remained under the GC *p* < 0.005 significance level. However, a new association was identified in chromosome 1 for the SNP rs1060622 (*TMED5*) (Table 2). None of the tested SNPs reached the genome-wide significance threshold when either the FDR or the Bonferroni correction were applied.

**Table 1:** Most relevant results (GC *p* < 0.005) of the transmission disequilibrium tests in the total cohort

Chromosome	SNP	Gene	Major allele > Minor allele	Odds ratio	95% CI		GC *p*-value
					Lower bound	Upper bound	
1	rs952499	*ABCA4*	C > T	4.50	1.52	13.30	0.003
3	rs1874925	*SYNPR*	C > T	0.16	0.05	0.53	0.002
4	rs724659	*LOC124900822*	T > C	0.34	0.17	0.71	0.005
5	rs261198	-	T > G	0.24	0.11	0.55	0.001
10	rs761774	*ADGRA1*	A > G	0.25	0.10	0.61	0.002
11	rs1488618	-	G > T	0.25	0.09	0.67	0.003
12	rs1486629	*TMEM132D*	G > A	0.25	0.10	0.61	0.001
13	rs3847993	*IL17D*	G > A	4.50	1.52	13.30	0.003
13	rs726455	*SOX1-OT*	C > T	5.00	1.45	17.27	0.005
14	rs742893	-	G > A	0.30	0.13	0.71	0.003
15	rs877228	*RORA*	T > C	0.28	0.13	0.63	0.004
20	rs1131382	*SLC23A2*	A > G	0.33	0.15	0.74	0.005
X	rs845324	-	A > G	0.24	0.08	0.70	0.004

**Table 2:** Most relevant results (GC *p* < 0.005) of the transmission disequilibrium tests in the cohort of participants without clefts

Chromosome	SNP	Gene	Major allele > Minor allele	Odds ratio	95% CI		GC *p*-value
					Lower bound	Upper bound	
1	rs1060622	*TMED5*	T > C	0.28	0.12	0.71	0.005
5	rs261198	-	T > G	0.26	0.11	0.64	0.003
12	rs1486629	*TMEM132D*	G > A	0.25	0.09	0.67	0.003
20	rs1131382	*SLC23A2*	A > G	0.25	0.09	0.67	0.003
X	rs845324	-	A > G	0.13	0.03	0.58	0.003

The prevalence of Class I malocclusion in our total cohort is 14.7%, where 50.5% of affected individuals are males and 49.5% are females. For the subset of individuals in the total cohort who have Class I malocclusion and not clefts, the prevalence of Class I malocclusion is 15.9%, where 49.5% are males and 50.5% are females. We broke down the GC significant genotyping results by sex in the total cohort and in the cohort of participants with Class I malocclusion without clefts and report the most relevant (GC *p* < 0.005) results in Table 3. The rs1874925 in *SYNPR* reached GC significance threshold in the total cohort with females more frequently carrying the homozygous for the major allele (CC) (Table 3). When breaking down the genotyping results by Class I status and comparing the cases and the comparison group, no significant associations were identified. Using the presence of oral conditions including dental caries as a covariate also did not affect the results identified.

**Table 3:** Most relevant (GC *p* < 0.005) results of the genotyping differences by sex in the patients from the total cohort and the without clefts cohort who were affected by Class I malocclusion

SNP/Gene	Total cohort		Without clefts cohort
	rs1874925/SYNPR	rs845324/Chr X	rs845324/Chr X
Sex			
Males	15 CC	26 A_	24 A_
	25 TC	0	0
	6 TT	20 G_	18 G_
Females	32 CC	16 AA	15 AA
	11 TC	27 AG	27 AG
	3 TT	3 GG	3 GG
Chi square *p*-value	0.002	N/A	N/A

N/A - We are not able to calculate a *p*-value since the rs845324 is in chromosome X

## Discussion

In the present study, we report various associations between SNPs within the genome and Class I malocclusion in participants with and without clefts which have not yet been examined in the literature. Two significant associations found in this study were for SNPs in the genes *ABCA4*, which encodes the membrane-associated protein ATP-binding cassette transporter [12], and *SOX1-OT*, which is a non-coding RNA that regulates gene expression [13]. Previous research showed that *ABCA4* is associated with craniofacial skeletal variation among patients with skeletal malocclusion [14], and many studies have found associations between *ABCA4* and *SOX1-OT* and cleft lip with or without cleft palate [15–19]. The associations of these genes with skeletal malocclusion and clefts in previous research and with dental malocclusion in our project, despite the fact that we are focusing on dental malocclusion rather than skeletal malocclusion, indicates that there may be a shared genetic basis for various aspects of craniofacial growth and development.

Another significant association identified in this study was for a SNP in the gene *RORA*, which encodes for the nuclear hormone receptor RAR related orphan receptor A [20]. This gene has been shown to be associated with a risk of orofacial clefts [21] and neuropathy in individuals with head and neck cancer [22]. *RORA* was also found to exhibit significantly decreased transcript expression in dental pulp stem cells from subjects with 15q Duplication syndrome compared to controls [23]. Individuals with this syndrome can have unusual facial features such as micrognathia, a high-arched palate, a long philtrum, and anteverted nares [24]. The *IL17D* association was a novel finding and there is a lack of literature describing implications related to this gene.

Additionally, we broke down the significant genotyping results by sex in the affected patients from the total cohort and identified significant differences in the frequency of the rs1874925 in *SYNPR* between males and females.

We are aware that the small sample size and the specificity of the Filipino cohort limit the generalizability of our study. We therefore need to confirm our findings using a larger sample and multiple populations that are exposed to various environmental influences. However, the cohort’s shared exposure to the same environmental factors during data collection reduced the heterogeneity of the sample, boosted confidence that the control group accurately represented the affected group, and increased the likelihood of discovering biologically relevant results. It is also relevant to note that three methods for correction were implemented: GC, FDR, and Bonferroni. Statistically significant results were found only when the genomic control correction [25] was used. This is likely due to the fact that GC controls for confounding effects of population stratification, based on the distribution of the chi-square statistics for the allele suspected to be associated with the phenotype compared to the same distribution of another allele not associated with the phenotype in a different chromosome. It is important to highlight that GC may be a too relaxed multiple testing approach and our results should be taken with caution since that false positives are a possibility.

Our study was not only limited by the small sample size but also the unremarkable age distribution of the participants (patients and family members were often young). It is necessary to corroborate our findings with the addition of age as a variable. Additionally, in future studies, it would be interesting to investigate the association between Class I malocclusion with the different subtypes of clefts. Since in our study only six people had both clefts and Class I malocclusion diagnoses, we could not accurately investigate this relationship.

In conclusion, this study adds to the body of knowledge on genetics of malocclusion and cleft lip and/or palate by showing that several SNPs previously linked to a variety of craniofacial phenotypes are linked to a Class I molar relationship. The notion of a shared genetic basis for disparate elements of craniofacial development is intriguing and further research should explore this possibility as well as the processes by which these genes may interact to cause the observed phenotypes.

## Data Availability

The datasets used and/or analyzed during the current study are available from the corresponding author on reasonable request.
